# Evaluation of the impact of fibromyalgia on patients' sleep and the content validity of two sleep scales

**DOI:** 10.1186/1477-7525-7-64

**Published:** 2009-07-10

**Authors:** Susan Martin, Arthi Chandran, Laurie Zografos, Gergana Zlateva

**Affiliations:** 1 RTI Health Solutions, 3040 Cornwallis Road, Research Triangle Park, NC, 27709, USA; 2 Pfizer Inc, 235 East 42nd Street, New York, NY, 10017, USA

## Abstract

**Background:**

Disturbed sleep is commonly reported in fibromyalgia (FM). Both the Sleep Quality Numeric Rating Scale (NRS) and the Medical Outcomes Study Sleep Scale (MOS-Sleep) have demonstrated positive psychometric properties in patients with FM. However, these assessments were developed prior to the current recommendation to include patient input during the concept elicitation or item generation phases. Therefore, the objective of this study was to evaluate the impact of FM on participants, including their sleep, and to test the content validity of these two sleep measures in FM patients.

**Methods:**

Qualitative interviews were conducted in Raleigh, North Carolina and Detroit, Michigan with 20 adults who reported a physician diagnosis of FM. Sixteen participants were female, 13 were white, and the average age was 50 years. Two researchers conducted all interviews using a structured guide.

**Results:**

Participants consistently reported that FM had a debilitating impact on their lives and their sleep, particularly getting to sleep and staying asleep. Participants responded positively to the Sleep Quality NRS as an assessment of their sleep. The majority of participants stated that they would not change the response numbering or wording of the item's anchors. Participants also responded positively to the 24-hour recall period of the Sleep Quality NRS. Participants found the 12-item MOS-Sleep to be appropriate and relevant; 19 participants indicated the measure captured all of their sleep-related symptoms. However, areas for potential modification were identified, such as the need to separate the item regarding awakening short of breath and awakening with a headache into two separate questions. Participants also questioned the relevance of the snoring and awakening short of breath items to FM. Half of participants expressed a preference for a daily rather than a weekly recall period.

**Conclusion:**

This study demonstrates the significant impact that FM has on patients' lives, particularly sleep. While patients with FM were not part of the development of the generic sleep assessments that were evaluated, this study provides evidence of their content validity, supporting their use in FM studies. Modifications to the MOS-Sleep may improve the psychometric properties and relevance to patients with FM.

## Background

Fibromyalgia (FM) is defined by the American College of Rheumatology (ACR) criteria as chronic widespread pain (present for at least 3 months) accompanied by pain upon digital palpitation in at least 11 of 18 defined tender point sites [1]. FM has an estimated prevalence of 2% of the United States adult general population, with a greater estimated prevalence among women (3.4%) than men (0.5%) [2]. While pain is the defining symptom of FM, commonly accompanying symptoms or conditions include fatigue, disturbed sleep, anxiety, depression, irritable bowel syndrome, headache, concentration or memory problems, and numbness/tingling sensations [1].

Qualitative studies have elicited the key symptoms of FM from the patient [3-14] and the clinician perspectives [14], with results consistently indicating that sleep disturbance plays a prominent role in the disease. Estimates of the percentage of FM patients experiencing some sleep problem range from 74% in the population on which the ACR criteria were established [14] to as high as 95% and 99% in two recent studies [15,16], Symptoms of disturbed sleep in FM also have been shown to predict increased levels of pain [15,16] and decreased levels of physical functioning [15]. Not surprisingly, both patients and clinicians have expressed the importance of targeting sleep for treatment improvements; only the symptoms of pain and fatigue received higher levels of endorsement in ranking exercises [14]. Thus, the accurate assessment of sleep changes associated with treatments for FM is of critical importance.

A wide range of sleep patient-reported outcome (PRO) measures have been used in the study of FM, including single-item numeric rating scales (NRS), visual analogue scales (VAS), and various multi-item scales [17]. Two such sleep measures, the Sleep Quality NRS and the Medical Outcomes Study Sleep Scale (MOS-Sleep) [18], have demonstrated strong psychometric properties, including reliability, validity, and responsiveness, [19-24] specifically in the FM patient population. Both of these PRO measures were developed prior to the current emphasis on the involvement of patients at the concept elicitation and item generation phases [25], and neither was originally intended as an FM-specific assessment tool. Therefore, the objectives of the current qualitative study were to further describe the impact of FM on participants' sleep and to evaluate the content validity of both of these measures in the FM patient population.

## Methods

### Participants

Eligible patients were between the ages of 18 and 65 years (inclusive), were diagnosed with FM for 6 months or more, and reported that FM affected their sleep. Exclusion criteria included the presence of other factors that could affect sleep, such as diagnosed sleep disorders; extenuating circumstances, such as a young child at home; or night-shift work. To further confirm the FM diagnosis, participants also responded to questions regarding the nature, frequency, and location of their pain. Recruitment took place in August and September of 2008 and for each round of interviews, efforts were made to recruit participants diverse in gender, race, and educational background.

### Procedures

The recruitment process for this study was initiated after RTI International's Institutional Review Board reviewed and approved implementation of the study, including the participant consent form and all of the materials used for recruitment. Participants for this study were identified by two qualitative research firms, which contacted individuals interested in participating in qualitative studies either through existing databases or Web advertisement. Trained recruiters screened all respondents in an effort to find individuals who were both eligible and interested in participating in the in-depth patient interviews.

Each interview was conducted by the same two researchers to ensure consistency of the questions and methods for each interaction. The interviews lasted approximately 1 hour and were initiated only after participants had completed an informed consent form. An interview guide, developed specifically for the study, was followed for each interview. The interviews started with a general discussion regarding the impact of FM on participants' lives and then moved to a discussion about the impact of FM on the participants' sleep. Following these open-ended queries, a cognitive debriefing of the two PRO measures was conducted [26]. Specifically, the participants were presented with each of the measures; participants were asked to complete each question using a "think-aloud" process [27,28] and answer follow-up probes from the interviewers related to the content, wording, and comprehension of the item. Participants were then asked to provide other feedback on the measures, such as their relevance to FM, and offer suggestions for improvement.

### Measures

The Sleep Quality NRS is a single-item measure that instructs the patient to "select the number that best describes the quality of your sleep during the past 24 hours," where 0 is best possible sleep and 10 is worst possible sleep (Table 1). The MOS-Sleep [18] is a 12-item measure that contains 7 subscales and 2 overall index scores (a 6-item and a 9-item index) (Table 2). The MOS-Sleep has demonstrated positive psychometric properties in a broad range of patient populations [18,29-32,23,24], including populations that, similar to those with FM, have chronic pain conditions that are accompanied by impaired sleep, such as postherpetic neuralgia [29], diabetic painful neuropathy [30], and rheumatoid arthritis [32].

**Table 1:** Sleep Quality Numeric Rating Scale

**Please complete the following question upon awakening. Select the number that best describes the quality of your sleep during the past 24 hours. (Circle one number only.)**										
0	1	2	3	4	5	6	7	8	9	10

**Best** **possible** **sleep**										**Worst** **possible** **sleep**

**Table 2:** MOS Sleep Scale Subscales and Scoring^a^

**Subscale**	**Number of Items**	**Brief Description (Item Number on Scale)**	**Scoring Range^b^**
Sleep disturbance	4	Trouble falling asleep (7); time to fall asleep (1); restless sleep (3); awaken during sleep (8)	0 to 100
Snoring	1	Snore during sleep (10)	0 to 100
Awaken short of breath or with headache	1	Awaken short of breath or with headache (5)	0 to 100
Quantity of sleep	1	Quantity of sleep (2)	0 to 24
Optimal sleep	1	"Optimal" sleep (2)^c^	0 or 1^c^
Sleep adequacy	2	Enough sleep to feel rested (4); amount of sleep needed (12)	0 to 100
Somnolence	3	Feel drowsy during day (6); trouble staying awake during day (9); take naps (11)	0 to 100
Sleep Problem Index II^d^	9	Trouble falling asleep (7); time to fall asleep (1); restless sleep (3); awaken during sleep (8); awaken short of breath or with headache (5); enough sleep to feel rested (4); amount of sleep needed (12); feel drowsy during day (6); trouble staying awake during day (9)	0 to 100

^a ^See Additional file 1 for full MOS-Sleep Scale.

^b ^Higher scores indicate more of the concept being measured (e.g., higher Sleep Disturbance scores indicate greater sleep disturbances). Quantity of Sleep scores are the patient-reported number of hours of sleep per night.

^c ^If the patient reports 7 or 8 hours of sleep per night, the Optimal Sleep score is 1; otherwise the Optimal Sleep score is 0.

^d ^Nine-item summary score, sometimes referred to as Sleep Index II. The 6-item summary scale (Sleep Index I) includes the same items, with the exception of item 1 (time to fall asleep), item 3 (restless sleep), and item 6 (feel drowsy during the day), and was developed as a briefer alternative to the 9-item index.

### Analysis

Interview results were compiled via audiotape and detailed notes recorded during each interview. No transcripts were created. Upon conclusion of the interviews, both forms of documentation (audio and written) were reviewed and analyzed to facilitate preparation of a comprehensive report. The report summarized findings on the overall impact of sleep on FM, as well as the specific findings and feedback on each PRO measure.

## Results and discussion

### Participant characteristics

Interviews were conducted in September 2008 in Detroit, Michigan and Raleigh, North Carolina. As shown in Table 3, 20 men and women aged 29 through 64 years, with an average age of 50.3 years, participated in the study. Across interviews, the sample was predominately white (65%) and female (80%). Participants reported having a diagnosis of FM for 8.9 years on average, and most of the participants reported that a rheumatologist (40%) or a primary care practitioner (35%) had initially diagnosed them with FM. Responses to the screening questions indicated that 18 of the participants experienced pain every day, and the remaining 2 participants experienced pain four to six times a week. Additionally, each of the participants reported pain in all four quadrants of their body, and their average typical pain level was 6.0 on a 0- to 10-point scale (0 indicating no pain and 10 indicating worst possible pain).

**Table 3:** Characteristics of cognitive interview participants (N = 20)

**Characteristic**	**Number (Percent)^a^**
**Gender**, n (%)	
Male	4 (20%)
Female	16 (80%)

**Age**, years, mean (range)	50.3 (29–64)

**Race^b^**, n (%)	
White	13 (65%)
Black	7 (35%)

**Education**, ^b ^n (%)	
Advanced degree	3 (15%)
College graduate	2 (10%)
Associates degree	2 (10%)
Some college	9 (45%)
High school degree or equivalent	4 (20%)

**Employment status**	
Employed full-time	5 (25%)
Employed part-time	1 (5%)
Unemployed	1 (5%)
Disabled	8 (40%)
Retired	3 (15%)
Other^b^	2 (10%)

**Years since diagnosis of FM**, mean (range)	8.9 (<1–18)

**Type of physician who diagnosed FM**	
Primary care practitioner	7 (35%)
Internal medicine specialist	2 (10%)
Pain specialist	1 (5%)
Rheumatologist	8 (40%)
Other^c^	2 (10%)

**Average (SD) of reported pain level^d^**	6.0 (1.6)

FM = fibromyalgia.

^a ^Unless otherwise noted.

^b ^One participant reported employment status as disabled and retired and is therefore counted in the Other category.

^c ^Two participants reported being diagnosed by multiple physicians and are therefore counted in the Other category.

^d ^On a 0–10-point scale where 0 indicates "no pain" and 10 indicates "worst possible pain." If participants provided two values (e.g., "6 or 7"), then the midpoint was used (e.g., 6.5).

### Impact of FM in general and specific to sleep

Each interview began with a general discussion of participants' experiences with FM and related symptoms. Participants provided a broad range of areas on which FM had a significant and negative impact, including their mood, family and social relationships, and work productivity. One participant stated that "it affects every aspect of your life" and that it was "very difficult to get relief from." Participants also discussed the need to push themselves to do activities and indicated that the fatigue they experience makes even simple tasks, such as taking a shower, difficult.

When discussing the impact of FM on their sleep, half of the participants reported difficulty getting to sleep due to FM, and all but one reported difficulty staying asleep due to FM. Those who had trouble getting to sleep commonly reported that the pain of FM made it difficult for them to get comfortable, and therefore, they often "tossed and turned" in their attempts to find a pain-free position for sleep. Participants who had difficulty staying asleep reported that they were frequently awakened during the night due to the pain of FM. Participants also spoke of no longer feeling refreshed upon awakening since having FM, regardless of the amount of sleep that they received.

Participants reported it was difficult for them to function after a poor night of sleep, stating that the next day they felt "groggy," "sluggish," or "foggy," and their mood and concentration were also affected. Similarly, several participants also commented on the cycle of increased pain and poor sleep; one participant described this cycle as "pain leads to a bad night, and a bad night leads to pain the next day."

### Cognitive debriefing results

#### Sleep Quality Numeric Rating Scale

##### Relevance for fibromyalgia

Fourteen of the participants responded positively when asked how well the Sleep Quality NRS captured the effect of FM on their sleep, including participants who stated the item was "perfect," the "best and most all-inclusive question you could ask," and captured "broad overall quality." When asked what aspects were missing from this question, five participants said that specific details regarding their sleep were not captured, such as the number of awakenings through the night, the ability to go to sleep, and difficulty getting up in the morning. Four participants said that other factors influencing sleep were missing, such as why the sleep had been poor and if medication had been taken. One participant thought that the term "fibromyalgia" should be included in the item, and two participants thought that issues not related to sleep, but related to FM, should be addressed, such as mood, appetite, degree of pain, and level of fatigue.

##### Interpretation of sleep quality

When asked to describe the concept in their own words, 14 participants used the words "restful," "rested," "not tired," or "refreshing" in their definitions of "sleep quality." Some participants also said the question was asking them "how well" they had slept, if they had slept through the night without awakening, and how much sleep they had gotten.

##### Ease of administration and response scale

Participants generally responded positively to the format of the Sleep Quality NRS, stating that it was easy to understand and complete, and several participants commented that it mirrored the way their physicians routinely inquired about their pain level. Regarding the 0 to 10 response scale, most participants (n = 14) indicated that they would not make any changes to the response numbering or the anchor wording. Three participants thought that the anchors should be switched so that 10 would correspond to the best possible sleep, because they associated higher numbers with a better outcome, and three participants thought that fewer responses, such as a 5-point scale, would be easier to use.

##### Recall period

Participants consistently said that remembering their sleep over the past 24 hours was easy to do. Participants indicated that they had been thinking of "last night" when they completed the item, and a few indicated that unless naps taken the prior day were of interest, that they would recommend stating "last night" instead of "the past 24 hours."

#### MOS-Sleep

##### Relevance for fibromyalgia

In general, participants found the measure to be relevant to their sleep symptoms, providing statements such as "sounds like me in a nutshell," and when asked about the item relevance, stating "all are relevant." However, participants did provide additional feedback when reviewing each of the individual items. For instance, many reported that they did not think that they "spoke in their sleep," which was one of the examples for sleep that was not "quiet" in item 3. Several participants also provided words that they thought would better reflect their sleep than "quiet" for item 3, such as "restful," "good," or "comfortable" sleep. Nearly every participant felt that item 5, "awakening short of breath or with headache," needed to be split into two questions if both concepts were retained. While many thought awakening with a headache was relevant for FM, most did not think awakening short of breath was relevant. The item on snoring (item 10) was rated as the least relevant overall, with 11 participants stating it was not important in FM.

When asked what aspects were missing from the MOS-Sleep, participants generally mentioned other symptoms or impacts of FM and not sleep-specific areas. For instance, participants stated that questions could be added about "anxiety," "mood," "confusion," "fatigue," "depression," or "joint pain." A few participants mentioned adding questions about medications or external circumstances that could affect sleep. The only suggestion that was specific to sleep symptoms was provided by a participant who stated that a question about "trouble waking up" or "getting out of bed" could be added.

To aid interpretation of the results for each measure, one supplemental question was added after completion of the fourth interview (and thus asked of only 16 of the 20 participants). The question asked participants, if only one of the two measures could be administered to FM patients, which should be chosen. Fifteen of the 16 participants stated that the MOS-Sleep should be chosen over the Sleep Quality NRS given the greater detail it provided about their sleep, an additional indication of the overall relevance of the measure from the participants' perspectives.

##### Ease of administration and response scales

Improvements to the format of the MOS-Sleep scale were identified by participants. Half of the participants suggested shortening the 6-point response scale; generally by recommending consolidation of one or two categories with similar verbal descriptions. For instance, four participants said they would eliminate "a good bit of the time" and a "little bit of the time," stating that they were too similar to "most of the time" and "some of the time," respectively. Additionally, an issue arose for four participants around the reversal of direction for MOS-Sleep item responses; these participants had difficulty answering item 4 because the directionality of the responses was reversed from item 3 [see Additional file 1].

##### Recall period and timing of administration

Ten of the participants stated that they would prefer a daily recall period versus the current 1-week recall period. Many of these participants noted that it was too difficult to remember and/or give an accurate answer over a period of a week.

## Discussion and conclusion

Similar to previous qualitative studies described in the literature, participants in this study expressed that FM had a profoundly negative impact on their lives in general and their sleep in particular. Many participants reported that since having FM, they were no longer able to experience a truly good night of sleep that would result in feeling refreshed or rested upon awakening.

Participants' responses to general, open-ended questions regarding the impact of FM on their sleep, as well as their feedback upon item review, provide strong evidence for the content validity of both PRO measures for complementary purposes. Specifically, the Sleep Quality NRS was reported by participants to be relevant and appropriate for addressing the overall impact of FM on their sleep. Furthermore, participants stated that the overall assessment of their sleep quality facilitated by this measure was both important and similar to the type of information they provided to their physicians about the consequences of their nocturnal FM pain. These findings, along with the brevity, ease of use, and daily recall period provide further support for use of the Sleep Quality NRS in both routine clinical practice and in research studies as part of a patient diary.

The MOS-Sleep was considered to be of similar relevance by the participants while providing a more comprehensive and detailed assessment of the impact of FM on their sleep. Nineteen of the participants said that the instrument captured all of their sleep-related symptoms, indicating likely concept saturation of these symptoms. However, participants also provided evidence that the MOS-Sleep could be modified to better fit patients with FM. Several implications for further research arise from these results. For example, eliminating "short of breath" or at a minimum, separating it from "headache" in item 5 and eliminating item 10 (snoring) may improve the measure's relevance and potentially its psychometric performance within the FM patient population. Both items were originally included to assess breathing-related sleep disorders in the general population. Additionally, evaluation of a shorter recall period and employing a 4- or 5-point versus a 6-point response scale also could prove valuable. Regarding the implications for routine clinical practice, while patients endorsed that the broader scope of assessment of the MOS-Sleep offered valuable insight in the sleep disturbance process associated with FM, the 12-item scale requires more time for administration and scoring and its use is likely to be limited to clinical research rather than routine clinical practice.

A few limitations should be considered in evaluating this study. One limitation is that participants had to experience sleep problems attributed to their FM symptoms to participate because the study evaluated sleep assessments. While concomitant sleep disturbance is reported for the vast majority of patients with FM, with some recent studies reporting rates as high as 95% and 99% [15,16], this study population is not representative of those FM patients who do not experience disturbed sleep that they attribute to their FM. Another important limitation of this study is that only two sleep assessments were evaluated by the participants; further work should assess other commonly used sleep assessments, such as the Pittsburgh Sleep Quality Index [33], from the perspective of this patient population. Nevertheless, this is the first effort to establish the relevance and appropriateness of specific sleep assessments directly by patients with FM. Furthermore, this study represents a pragmatic, research-based approach to assess the content validity of PRO measures developed prior to the current development standards of patient involvement in the concept elicitation and item generation phases. The results reported here support and extend prior psychometric evidence supporting the use of these two measures in the FM patient population.

## Competing interests

The study was funded by Pfizer. Arthi Chandran and Gergana Zlateva are employed by Pfizer. Susan Martin and Laurie Zografos are employees of RTI Health Solutions, who were paid consultants to Pfizer in connection with the development of this manuscript.

## Authors' contributions

SM contributed to the study design, data collection, interpretation, and analysis of results and manuscript creation. AC contributed to the study design, analysis of results, and manuscript review. LZ contributed to the study design, data collection, interpretation, and analysis of results and manuscript review. GZ contributed to the study design and manuscript review. All authors have read and approved the final manuscript.

## Supplementary Material

Additional file 1 **MOS- Sleep Scale**. The data provided represent the full instrument version of the MOS-Sleep Scale, including appropriate copyright information. This document is also publically available at the following URL: . Although already publically available, the authors have also contacted the copyright holders for permission to reproduce as an appendix to this article. Click here for file
