# Supplementary material for: Evaluation of the impact of fibromyalgia on patients' sleep and the content validity of two sleep scales
Source: Health Qual Life Outcomes. 2009 Jul 10;7:64. doi: 10.1186/1477-7525-7-64 (PMC2717926; doi:10.1186/1477-7525-7-64)
Supplement: Additional file 1 — MOS- Sleep Scale. The data provided represent the full instrument version of the MOS-Sleep Scale, including appropriate copyright information. This document is also publically available at the following URL: . Although already publically available, the authors have also contacted the copyright holders for permission to reproduce as an appendix to this article. [file 1477-7525-7-64-S1.pdf]

Sleep Scale from the Medical Outcomes Study

1. How long did it usually take for you to fall asleep during the past 1week?

(Circle One)

0-15 minutes…………..…1

16-30 minutes……………..2

31-45 minutes……………..3

46-60 minutes……………..4

More than 60 minutes …….5

---

2. On the average, how many hours did you sleep each night during the past 1 week?

Write in number
of hours per night: ☐☐

How often during the past 1 week did you…

(Circle One Number On Each Line)

	All of the Time	Most of the Time	A Good Bit of the Time	Some of the Time	A Little of the Time	None of the Time
3. feel that your sleep was not quiet (moving restlessly, feeling tense, speaking, etc., while sleeping)?	1	2	3	4	5	6
4. get enough sleep to feel rested upon waking in the morning?	1	2	3	4	5	6
5. awaken short of breath or with a headache?	1	2	3	4	5	6
6. feel drowsy or sleepy during the day?	1	2	3	4	5	6
7. have trouble falling asleep?	1	2	3	4	5	6
8. awaken during your sleep time and have trouble falling asleep again?	1	2	3	4	5	6
9. have trouble staying awake during the day?	1	2	3	4	5	6
10. snore during your sleep?	1	2	3	4	5	6
11. take naps (5 minutes or longer) during the day?	1	2	3	4	5	6
12. get the amount of sleep you needed?	1	2	3	4	5	6

Copyright, 1986, RAND

Hays, R. D., & Stewart, A. L. (1992). Sleep measures. In A. L. Stewart & J. E. Ware (eds.), Measuring functioning and well-being: The Medical Outcomes Study approach (pp. 235-259), Durham, NC: Duke University Press.
